# Molecular Stress Response of Mitochondria during Electrostimulation Evoking Stem Cell Differentiation Revealed by Fluorescence Imaging Combined with SERS Spectra

**DOI:** 10.1021/acsmeasuresciau.5c00005

**Published:** 2025-03-12

**Authors:** Jiafeng Wang, Xiaozhang Qu, Zhimin Zhang, Xiuping Meng, Guohua Qi

**Affiliations:** † Department of Endodontics, Hospital of Stomatology, 12510 Jilin University, Changchun 130021 Jilin, P. R. China; ‡ Guangdong Key Laboratory of Biomedical Measurements and Ultrasound Imaging, School of Biomedical Engineering, Shenzhen University Medical School, Shenzhen University, Shenzhen 518060, China; § 117971 The First Hospital of Jilin University, Changchun 130031, P. R. China; ∥ State Key Laboratory of Electroanalytical Chemistry, Changchun Institute of Applied Chemistry, 58277 Chinese Academy of Sciences, Changchun 130022 Jilin, P. R. China

**Keywords:** impulse electrical stimulation, dental pulp stem cell, cell differentiation, mitochondria, SERS spectra

## Abstract

Stem cells are a class of multipotential cells with the capability of self-replication, which can differentiate into multiple functional cells under extra stimulus. The differentiation of stem cells has important implications for tissue regeneration. Therefore, controllable regulation of dental pulp stem cell (DPSC) behaviors is critical for repairment and regeneration of damaged teeth tissues. Rapid promotion of DPSCs, directed differentiation, and revealing molecular events within the organelle level during the cell differentiation process are in great demand for regeneration of teeth, which remains a great challenge. Herein, we developed a highly effective and uncomplicated stimulation platform to promote the DPSCs for odontogenic differentiation based on impulse electrical stimulation and revealed the molecular stress response of mitochondria during cell differentiation based on fluorescence imaging combined with surface-enhanced Raman spectroscopy (SERS). Our approach can greatly shorten the DPSC differentiation time from usually more than 20 days to only about 3 days under 0.8 V for 5 min every day than drug stimulation. Notably, the glycogen and adenosine triphosphate levels within mitochondria were apparently elevated, which is conducive to improving the progression of cell differentiation. Simultaneously, the expression of mitofusin1 and mitofusin2 within mitochondria was significantly down-regulated during the differentiation process. Mechanistically, the molecular insights into mitochondria within DPSCs were clearly revealed through SERS spectra. It demonstrated that the expression of phenylalanine was significantly reduced, whereas the contents of tryptophan within mitochondria were promoted during the cell differentiation process. This study provides a comprehensive and clinically feasible strategy for the rapid promotion of DPSCs-directed differentiation and reveals the molecular dynamic changes within mitochondria, which broadens the biomedical cognition of electrical stimulation for dental pulp stem cell differentiation and provides a potential application for teeth tissue regeneration in the future.

## Graphical abstract

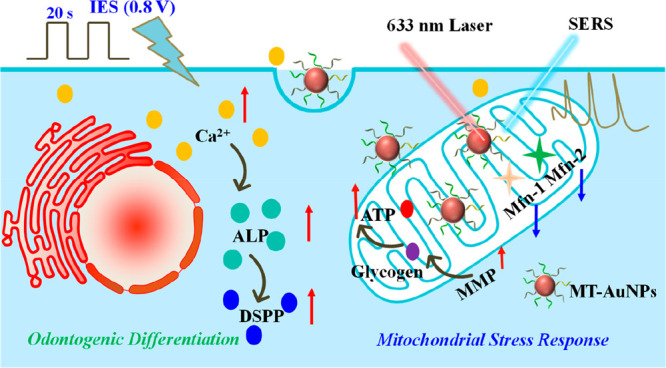

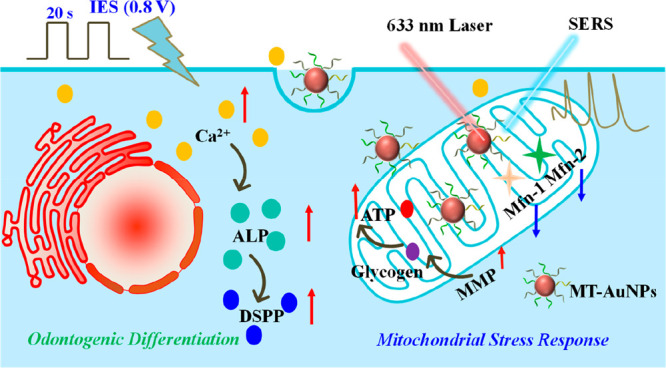

## Introduction

Stem cells are considered a promising treatment strategy for regenerative medicine because they can enable differentiation into specialized cells to form tissues.^1–4^ Dental pulp stem cells (DPSCs) as a population of adult stem cells are readily available, high cryopreservation capacity without losing their differentiation capability for a long period.^5,6^ Of note, the DPSCs possess self-renewal ability, strong proliferation, multidirectional differentiation, and so on.^7^ Additionally, adjusting DPSC differentiation into specific cell types is critical for pulp restoration and tooth regeneration. Consequently, the development of effective approaches is significant for regulating DPSC differentiation.

At present, drug stimulation is the traditional treatment method for regulating DPSC differentiation.^8^ However, it often takes a long time, until 21 days, for the stimulation of cell differentiation. To quickly promote dental pulp stem cell differentiation, some scaffold materials are designed and used to stimulate cell differentiation under extra physical conditions.^9,10^ Although these approaches can accelerate the progression of cell differentiation, some important issues still need to be addressed, such as complex material synthesis, material metabolism, and other side effects. Furthermore, acquiring a reproducible and controlled approach to guide DPSCs differentiation is the most critical concern. Therefore, it is indispensable to develop a simple, rapid, controllable, noninvasive, and reproducible methodology for promoting DPSC differentiation. To address these limitations, we developed the impulse electrical stimulation (IES) platform for DPSC differentiation due to the palpable superiority of IES, such as simple, stable, reproducible operation and good controllability. In this study, we found that the IES employed can quickly promote dental pulp stem cell-directed differentiation. Notably, understanding the relevant molecular biology information is also very important for DPSC differentiation during the electrical stimulation process.

Mitochondria, as highly dynamic organelles, are an important site for the energy generation of adenosine triphosphate (ATP).^11,12^ Simultaneously, the mitochondria participate in multiple life activities such as cell proliferation, aging, cell death, and differentiation reprogramming.^13^ The dynamic expression levels of key factors within mitochondria are vital for cellular function and tissue formation. To our knowledge, dynamic biomolecular information changes within mitochondria have not been reported during the DPSC differentiation process induced by IES. Consequently, the development of a new method for tracing the varieties of key factors and revealing molecular profiling of mitochondria is highly desired to understand the DPSC differentiation during the IES process.

Currently, the measurements of key intracellular molecules during cell differentiation are mainly through Western blot, gel electrophoresis, polymerase chain reaction, and mass spectra. These methods require cell lysis, biomolecular extraction, and purification, the operating steps of which were more complicated and time-consuming. Importantly, these traditional methods cannot acquire the in situ dynamic changes of biomolecules within cells. Cell fluorescence imaging possessed superiority, such as high specificity, dynamic real-time observation, noninvasiveness, and so on.^14,15^ However, cell fluorescence imaging can only label and measure a specific target and cannot detect substance changes of unknown complex components. It also cannot reveal molecular structural information changes. Notably, surface-enhanced Raman spectroscopy (SERS) possesses the excellent sensitivity and molecular specificity as well as the capability of detection for complex biological compositions.^16–21^ Consequently, the SERS method is widely applied in the biomedical field, which has garnered more and more attention to the measurement of key factor levels within cells, such as reactive oxygen species, pH, gaseous content, and so on.^22–25^ Meanwhile, El-Sayed and coworkers^26^ have revealed the molecular profiling within the cell nucleus from single cells during the cell apoptosis process based on SERS spectra. Importantly, Choi and co-workers have reported a 3D graphene oxide-encapsulated gold nanoparticle that is very effective for the detection of the differentiation potential of neural stem cells by SERS spectra.^27^ However, visualization imaging of a specific target by SERS spectra is slower than fluorescence imaging. Therefore, the fluorescence imaging combined with the SERS spectrum provides in situ and visual imaging of key biomolecules within cells and elucidates the molecular profiling of cells.

Herein, we present the uncomplicated and controllable methodology to promote the DPSC differentiation through IES (∼1 V applied bias), which shortens the differentiation time from customarily greater than 20 days of drug stimulation to only 3 days. It should be noted that the IES can boost the DPSC-directed differentiation toward odontogenic, which has high biological safety. Simultaneously, the key factors and associated molecular events within mitochondria during the DPSC differentiation process were revealed using fluorescence imaging combined with label-free SERS spectra, as shown in Scheme 1. The findings demonstrated that the upper elevation of the mitochondrial membrane potential (MMP) contributes to ATP generation and expression levels of mitofusin1­(Mfn-1) and mitofusin2 (Mfn-2) were significant down-regulation. Simultaneously, the fingerprint spectra of mitochondria within DPSCs during the cell differentiation process were revealed, which was that the contents of tryptophan were boosted and the phenylalanine levels were reduced. Overall, the feasible and controllable strategy for regulation of DPSC-directed differentiation was developed through IES, and molecular profiling of mitochondria within DPSCs was fully revealed, which broadens the cognition of stem cell differentiation and will possess potential application value for tooth regeneration in the future.

**Scheme 1:**
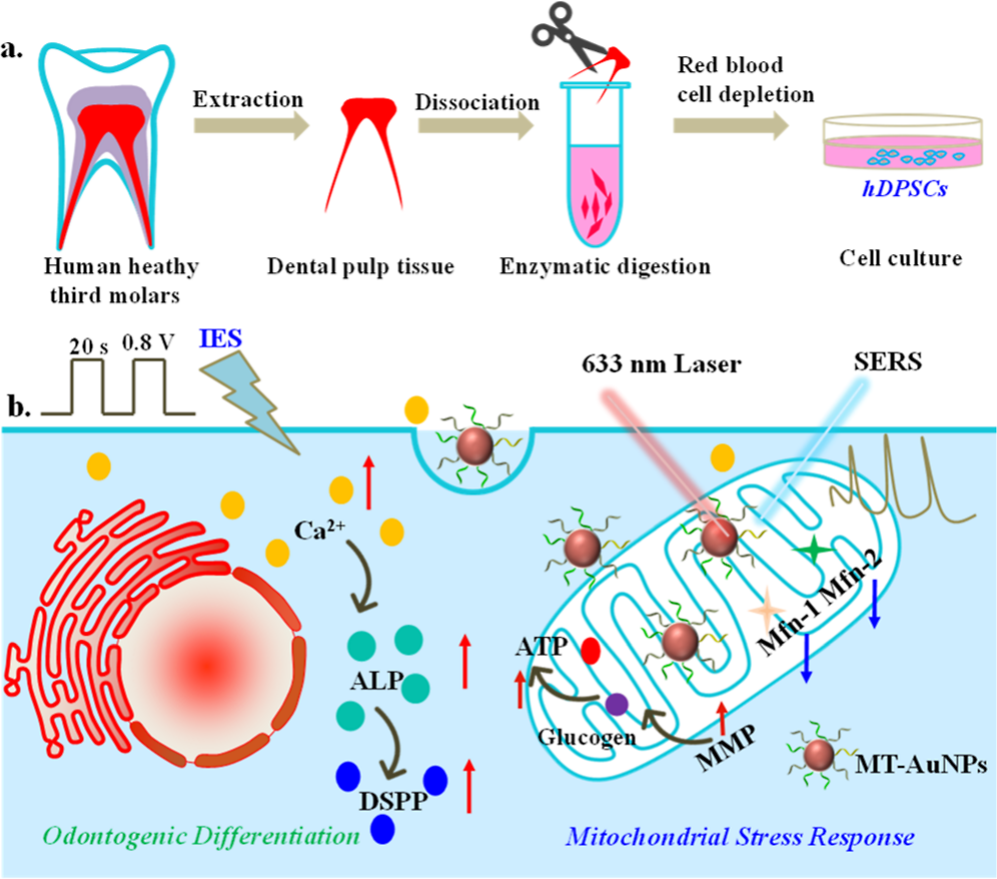
Schematic Illustration of Human Dental Pulp Stem Cell Extraction (a,b) Directing DPSC Differentiation by IES and Revealing Molecular Insights Based on Fluorescence Imaging Combined with Label-Free SERS Spectra

## Materials and Methods

### Materials

Sodium citrate (C_6_H_5_Na_3_O_7_) and Gold­(III) chloride trihydrate and trisodium citrate (98%) were purchased from Aladdin (Shanghai, China). The ATP assay kit (BC0305) and Ca^2+^ assay kit (F8840) were purchased from Solarbio. The sodium citrate, FITC labeled with dentin sialophosphoprotein (DSPP) antibody (LFMb-21), calcein-AM/propidium iodide (PI), alizarin red, alkaline phosphatase (ALP) (A14353) assay kit, and mercaphydryl polyethylene glycol (PEG, *M* = 5000) were obtained from Sigma-Aldrich. 3-(4,5)-Dimethylthiazo­(-z-y1)-3,5-diphenyltetrazolium bromide and dimethyl sulfoxide and JC-1 assay kit were bought from Key-GenBioTech. Sodium chloride, paraformaldehyde, Triton-X 100, and BSA were obtained from Aladdin. Dulbecco’s modified Eagle’s medium (DMEM), 0.25% trypsin/2.2 mM EDTA solution, antibiotic solution, and certified fetal bovine serum were bought from Vivacell, Shanghai. Antimitofusin 2­(ab56889) was obtained from Abcam. Antimitofusin 1­(A9880) was purchased from ABclonal. Cell-penetrating peptide (RGD) (RGDRGDRGDRGDPGC) and the mitochondria localization signal peptide (MLS) (MLALLGWWWFFSRKKC) were obtained from the Shanghai Apeptide Co., Ltd. The ultrapure water was obtained using a Millipore Milli-Q water purification system with an electric resistance >18.25 MΩ.

### Instrument

The transmission electron microscope (TEM) images of AuNPs were characterized through a Hitachi 600 TEM (Hitachi, Japan). The UV–vis spectra were recorded using a UV-2600 spectrophotometer (Shimadzu, Japan). The concentration of the AuNPs was detected by an ICAP 6300 inductively coupled plasma emission spectrometer (Thermo Fisher, USA). The bright and fluorescence images were collected using an inverted DMI6000B microscope (Leica, Germany). The zeta potential of nanoparticles was measured by a Zetasizer Nano ZS 90 (Malvern, British). The SERS spectra were recorded through a confocal Raman system (LabRAM ARAMIS, HORIBA Jobin Yvon).

### Regulating Human Dental Pulp Stem Cell Differentiation through Impulse Electrical Stimulation

Typically, the DPSCs (2 × 10^4^ cells) were seeded on conductive glass for overnight incubation. Then, the DPSCs were treated with IES. The ITO glass covered with cells was set as a working electrode. The reference electrode was Ag/AgCl (KCl saturated), and the counter electrode was a Pt sheet. In the IES process, the impulse voltages were optimized from 0.2 to 1.0 V under the same impulse width for 5 min. Meanwhile, the different pulse widths (5, 10, 15, 20, and 30 s) were measured for DPSCs under 0.8 V for 5 min. After IES for DPSCs under different conditions, the cleaned cells were cultured for 24 h at 37 °C in a humidified atmosphere containing 5% CO_2_. After that, the cells were stimulated under the IES on different days to check DPSC differentiation.

### Alkaline Phosphatase Expression within DPSCs after IES during the Differentiation Process

First, DPSCs were stimulated using the IES at 0.8 V for 5 min under an impulse width of 20 s per day with varied days. After that, PBS was used to clean cells three times. The ALP assay kit was added into DMEM complete medium as per the instruction to stain with DPSCs in the dark for 30 min. Subsequently, the cells were rinsed through PBS three times. Ultimately, the ALP fluorescent image within DPSCs was collected with the fluorescence microscope.

### Ca^2+^ Level within DPSCs during the Differentiation Process Induced by IES

To detect the Ca^2+^ level within DPSCs under different differentiation days after IES, the commercialized Ca^2+^ assay kit was used in this study. First, the DPSCs were cultured on the ITO glass and then stimulated through IES at 0.8 V for 5 min under a pulse width of 20 s for different days. The cells were cleaned with PBS and then stained using the Ca^2+^ assay kit for 30 min. Finally, the DPSCs were washed using PBS and recorded with a fluorescence detector of a Leica DMI6000B microscope with 10× objective.

### Adenosine Triphosphate Expression within DPSCs Stimulated by IES at Different Days

The ATP assay kit was used to check the ATP level within DPSCs during cell differentiation. Briefly, the DPSCs were treated with IES at 0.8 V for 5 min per day on different days (0, 1, 2, and 3 days). The same number of DPSCs was collected to detect ATP level changes. The cells were collected in centrifuge tubes to remove the supernatant. The ATP levels in cells were tested based on the operating instruction.

### Expression of Mitochondrial Fusion Protein within DPSCs under Different Cell Differentiation Days

The DPSCs were fixed with the paraformaldehyde (4 wt %) for 20 min and then cleaned through PBS. Subsequently, the cells were incubated with Triton-X 100 (1 wt %) for 20 min. The cells washed were treated with BSA (1 wt %) for 1 h. Thereafter, the antibody solutions of Mfn-1 or Mfn-2 were added into the PBS solution (at a dilution of 2:1000) for incubation with DPSCs at 4 °C overnight, respectively. The next day, the cleaned cells were stained with goat antirabbit lgG H&L (Alexa Fluor488) or Cy3-conjugated sheep antimouse secondary antibody (at a dilution of 2:500) for 1 h. After that, the cells were washed and then dyed with DAPI (1 μM) for 20 min. Finally, the fluorescence images of DPSCs were checked by the fluorescence microscope with a 20× objective.

## Results and Discussion

### Regulation of DPSC Differentiation through Impulse Electrical Stimulation

To investigate whether the IES can promote the rapid differentiation of DPSCs, the impulse voltages and widths were optimized under different conditions. As shown in Figure S1, the impulse currents were gradually boosted with the voltages increasing from 0.2 to 1.0 V for 5 min each day, which were used to treat with DPSCs. Simultaneously, the charges of cell electrodes were also amplified with voltage boosting (Figure 1a). Notably, the good cell viability of DPSCs was discovered from fluorescence imaging after IES treatment under different voltages for 2 days, as depicted in Figure S2. To estimate the effects of IES on the osteogenic differentiation of DPSCs under different voltages, the alizarin red staining was performed to assess the later stages of odontogenic/osteogenic differentiation with varied days (0, 1, and 2 days). As shown in Figure S3, the results showed that more calcium nodules were visible from DPSCs after the IES at 0.8 V for 2 days than other voltages. Similarly, we also estimated the effect of cell differentiation under different impulse widths for treatment of DPSCs. The impulse currents and charges were exhibited under different impulse widths (5, 10, 15, 20, and 30 s) at 0.8 V for 5 min, as shown in Figures 1b and S4. The results manifested that the obvious calcium nodules were found within DPSCs after treatment with IES at 0.8 V for 5 min under an impulse width of 20 s per day for 2 days (Figure 1c). Furthermore, the good biocompatibility of DPSCs after IES under different impulse widths was affirmed through fluorescent imaging through calcein-AM/propidium iodide (PI) (AM/PI) (Figure 1d). Consequently, the optimized impulse voltage and width are 0.8 V and 20 s, respectively, which are selected for regulation of DPSC differentiation in this work.

**Figure 1:**
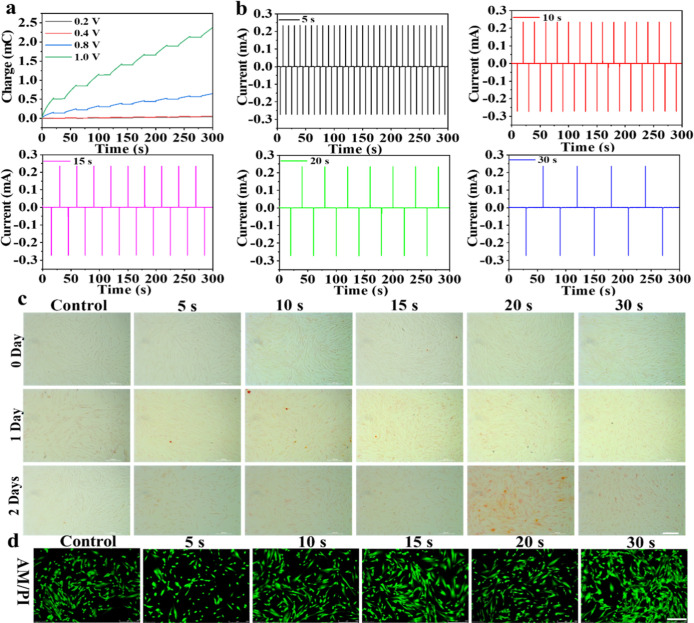
(a) Charge–time curves of DPSC electrodes after different voltages (0.2, 0.4, 0.8, and 1.0 V) stimulation for 5 min at 20 s of impulse widths. (b) Impulse current–time curves under different impulse widths (5, 10, 15, 20, and 30 s) after treatment of DPSCs at 0.8 V for 5 min. (c) Bright-field images of DPSCs stained with alizarin red after IES at 0.8 V under different impulse widths for 5 min on different days. The scale bar is 200 μm. (d) Fluorescence imaging of DPSCs stained with calcein-AM (4 μM)/propidium iodide (PI, 8 μM) for estimating cell activity after IES at 0.8 V for 5 min under different impulse widths for 3 days. The scale bar is 250 μm.

### Dental Pulp Stem Cell Differentiation toward the Osteogenic/Odontogenic Direction after IES

To estimate the feasibility of IES for the modulation of cell differentiation, the DPSCs were covered on the surface of conductive glass (ITO glass) and treated with IES for 3 days, as displayed in Figure 2a. Remarkably, this approach possesses good biocompatibility owing to the obvious green fluorescence imaging of DPSCs stained with the AM/PI assay kit (Figure 2b), even for the cells after IES treatment for 3 days. It is well-known that the dental pulp stem cells have the potential for multidirectional differentiation.^6^ Consequently, the two typical directions of differentiation are checked for adipogenic and odontogenic differentiation by the IES in this work. The oil red staining was used to examine the adipogenic differentiation of DPSCs after IES treatment, which is typically applied for the identification of cell adipogenic differentiation. As shown in Figure 2c, the DPSCs were not stained with oil red during the cell differentiation process, which implied that the IES cannot adjust the DPSCs toward the adipogenic direction. Attractively, more and more calcium nodules were observed with differentiation time lengthened through the alizarin red staining of DPSCs (Figure 2d). It indicates that the IES can promote the calcium nodule expression from DPSCs toward osteogenic differentiation. As displayed in Figure S5, the expression levels of ALP with DPSCs during the cell differentiation process are examined using the commercialized assay kit (1 μM) staining for 30 min and cleaning by PBS before observing, which is an important biomarker to assess the odontogenic differentiation.^8^ The significantly higher ALP activity within DPSCs after IES for 3 days was observed due to the stronger green fluorescence intensity than that of other groups. Furthermore, the associated fluorescence intensity of ALP within DPSCs during different cell differentiation days was then calculated, as shown in Figure 2e, which were 2.48 ± 1.2, 13.4 ± 1.6, 27.2 ± 3.3, and 45.7 ± 6.6, respectively. Quantitative analysis of ALP fluorescence imaging within DPSCs also confirmed that the expression of ALP was gradually increased with prolonged differentiation time. Simultaneously, the Ca^2+^ levels within DPSCs during the cell differentiation process were also assessed through a calcium-sensitive dye (Figure S6), which is considered the significant factor of osteogenic differentiation. The fluorescence imaging and quantitative analysis of fluorescence intensity of Ca^2+^ within DPSCs have indicated that the Ca^2+^ levels are evident up-regulation after IES treatment with differentiation time prolonged (Figures S6 and 2f). Furthermore, the DSPP is treated as another important odontogenic differentiation biomarker, which is secreted mainly from odontoblast.^28,29^ Therefore, expression levels of DSPP within DPSCs were determined through immunofluorescence staining during the cell differentiation process, as shown in Figure 2g. The results indicated that the DSPP contents were gradually boosted with the extension of the differentiation time. The final values of DSPP fluorescence intensity calculated were 4.69 ± 1.1, 10.2 ± 0.83, 28.4 ± 2.1, and 41.4 ± 3.0, respectively, at different differentiation times after IES (Figure 2h). Notably, the fluorescence intensity of DSPP within DPSCs without any stimulation (control group) was calculated over different days (Figure S7). The results manifested that the electrical stimulation can promote DSPP expression within dental pulp stem cells, compared with control groups. All these results have adequately affirmed that the IES can promote the DPSC differentiation toward the odontogenic direction for dentin. Subsequently, the cell length distributions of DPSCs were measured during the cell differentiation process, as displayed in Figure S8. As displayed in Figure 2i, the average length of DPSCs at different days was 79.4 ± 14, 106 ± 28, 127 ± 23, and 129 ± 29 μm, respectively. Meanwhile, the IES approach can greatly shorten the DPSC differentiation time to only about 3 days compared to traditional methods, as shown in Table S1.

**Figure 2:**
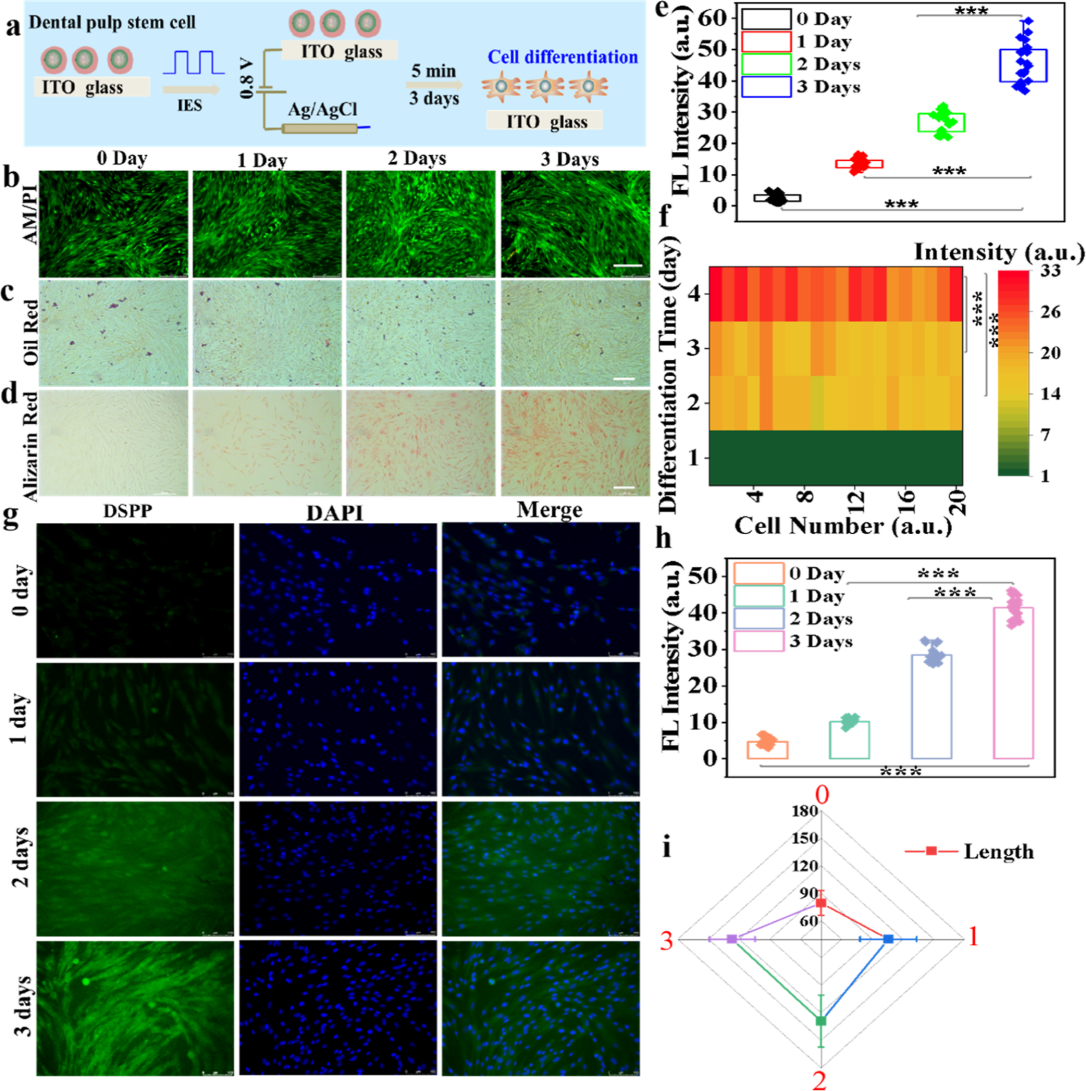
(a) Schematic diagram showing the regulation of the DPSC differentiation process based on IES. The fluorescence imaging (b) and bright-field images (c) of DPSCs stained with AM/PI and oil red after IES treatment with 0.8 V for 5 min at 20 s of impulse width under different days, respectively. (d) Bright-field images of DPSCs stained with alizarin red during the differentiation process by IES. All the scale bars are 100 μm. (e) Average fluorescence intensity of ALP within DPSCs after IES treatment at 0.8 V for 5 min on different days during the differentiation process. (f) Fluorescence intensity of Ca^2+^ within DPSCs heat map during the differentiation process after IES. (g) Immunofluorescence imaging of dentin sialophosphoprotein (DSPP) within DPSCs stimulated with IES under different days. The cell nucleus was stained with nuclear dye of 4′,6-diamidino-2-phenylindole (DAPI) (1 μM); the scale bar is 100 μm. (h) Fluorescence intensity of DSPP within DPSCs during the cell differentiation process, calculated using the software of Image-J. ***P < 0.0001, representing the statistical significance, which was calculated under the two-tailed Student’s *t*-test. (i) Average length changes of DPSCs after IES treatment under different days.

### Dynamic Response of Mitochondria within DPSCs during the Cell Differentiation Process

Mitochondria, as an important organelle, participate in significant life activities, including cell proliferation, apoptosis, and cell differentiation.^30^ In this work, the dynamic expressions of some key factors within mitochondria were investigated during the DPSC cell differentiation process. Typically, promoting the ATP generation within cells is based on the direct chemical driving force of MMP.^31^ Consequently, the MMP (Δψm) transformations within DPSCs during the cell differentiation process were checked using the commercialized dye of JC-1, as shown in Figure 3a. When JC-1 is concentrated in mitochondria, the red fluorescence (aggregation state) emerges, which indicates the mitochondria with high Δψm. While it releases into the cytoplasm, the green fluorescence (monomer state) is emitted to reflect the low Δψm of mitochondria. As depicted in Figure 3a, the green fluorescence intensity was gradually reduced within DPSCs, while the red fluorescence intensity was boosted with increasing differentiation days. Simultaneously, the fluorescence intensity ratios of the JC-1 aggregates and JC-1 monomers were calculated during the DPSC differentiation process, as shown in Figure S9a, which were 0.859 ± 0.084, 2.16 ± 0.32, 2.48 ± 0.38, and 3.2 ± 0.54, respectively. Simultaneously, the MMP of DPSCs in control groups (without any stimulation) was also measured (Figure S9b,c), the results of which manifested that MMP within DPSCs without any stimulation has slightly changed during different days, compared with IES groups. Thereafter, the ATP levels within DPSCs were estimated during the cell differentiation process in two groups (Figure 3b). It demonstrated that the ATP levels within cells were obviously increased with differentiation time prolonged after IES, compared with control groups. All these results have demonstrated that the IES can promote MMP within DPSCs elevated to contribute to the ATP generation, which further accelerates the rapid DPSC differentiation. In addition, the expression levels of mitofusin1­(Mfn-1) and mitofusin2 (Mfn-2) within DPSCs during the cell differentiation process after IES were performed through immunofluorescence imaging, which participates in the cell differentiation modulation.^32,33^ The expression level of Mfn-1 within DPSCs after IES was more palpable down-regulation on the third day of cell differentiation (Figure 3c,d), in contrast to undifferentiated DPSCs. Astoundingly, the Mfn-1 contents within DPSCs basically remain the same in the control groups on different days, as shown in Figure S10. Similarly, the Mfn-2 contents within cells after IES were more distinctly decreased than in control groups on the third day through fluorescence imaging and quantitative fluorescence intensity analysis, as shown in Figures S11, S12, and 3e. Notably, it implied that the degree of mitochondrial fusion was reduced during IES for DPSC differentiation.

**Figure 3:**
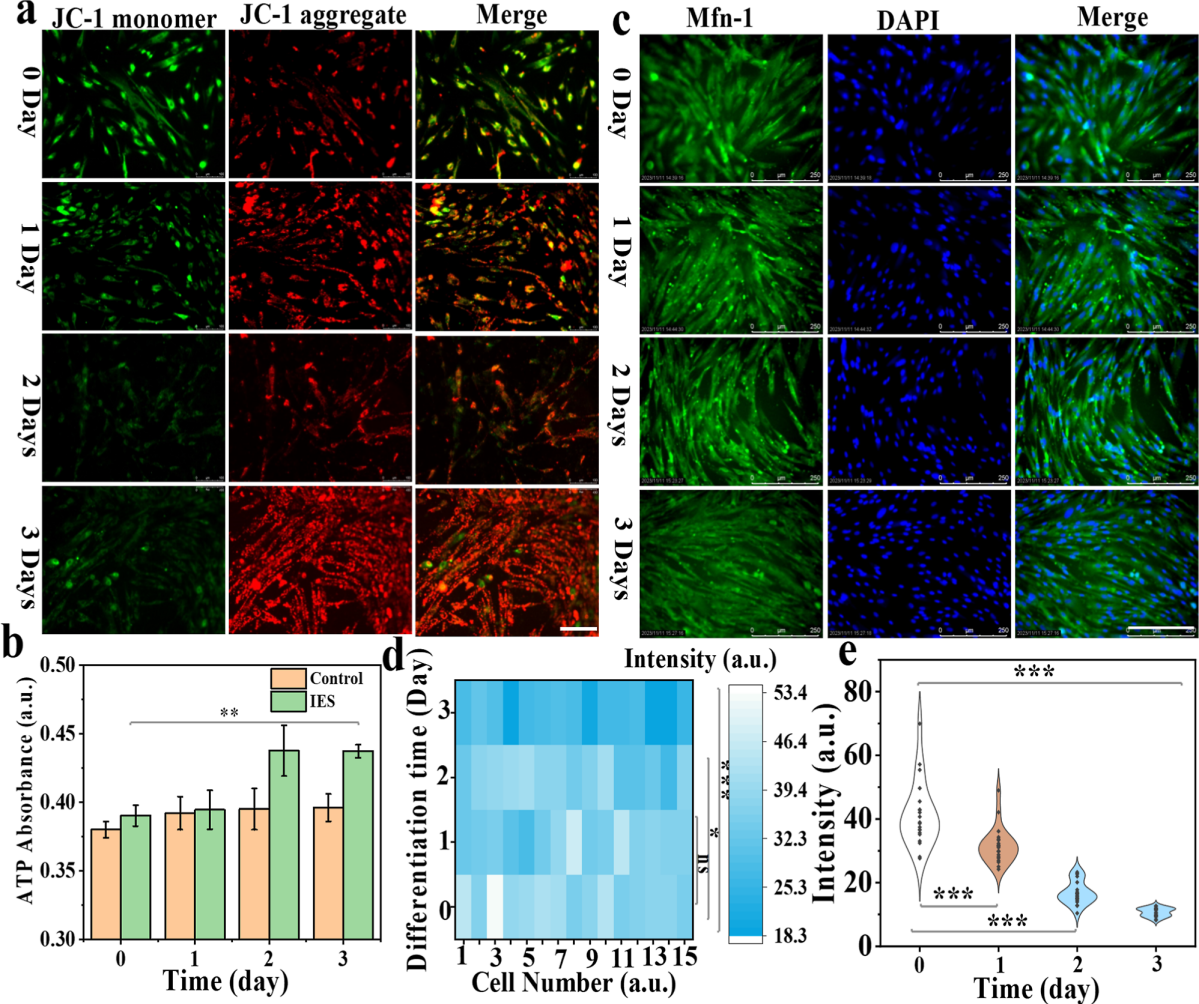
(a) Fluorescence images of MMP within DPSCs cells stained with JC-1 during the cell differentiation process (0, 1, 2, and 3 days) in IES groups. The scale bar is 100 μm. (b) ATP content changes within DPSCs after IES during different differentiation days. (c) Immunofluorescence imaging of Mfn-1 within DPSCs treated with IES under different differentiation days. The cell nucleus was stained with DAPI (1 μM). The scale bar is 250 μm. (d,e) Quantitative fluorescence intensity analysis of Mfn-1 and Mfn-2 within DPSCs after IES treatment during the differentiation process. *P < 0.05, **P < 0.001, and **P < 0.005 represent the statistical significance.

### Characterization of Mitochondrial Targeting Nanoprobes

To reveal the molecular profiling of mitochondria, the targeting nanoprobes were fabricated by the surface modification method,^34^ as shown in Figure 4a. The morphology of AuNPs was basically spherical from the TEM image (Figure 4b). The average diameter of AuNPs was 28.8 ± 4.8 nm from size distribution, as shown in Figure S13. As displayed in Figure 4c, the local surface plasmon absorption peaks of AuNPs appeared to have an obvious redshift from 528 to 535 nm owing to targeting peptide modification on the surface of AuNPs. Meanwhile, the surface charges of AuNPs have significant changes during the modification process (Figure 4d). The results manifested that the mitochondrial targeting nanoprobes (AuNPs&PEG&MLS&RGD, as MT-AuNPs) were successfully decorated in this work. Importantly, the good biocompatibility of mitochondrial targeting nanoprobes was affirmed using the standardized MTT assay when the concentrations of nanoprobes were less than 16.5 ppm for incubation with DPSCs (Figure 4e).

**Figure 4:**
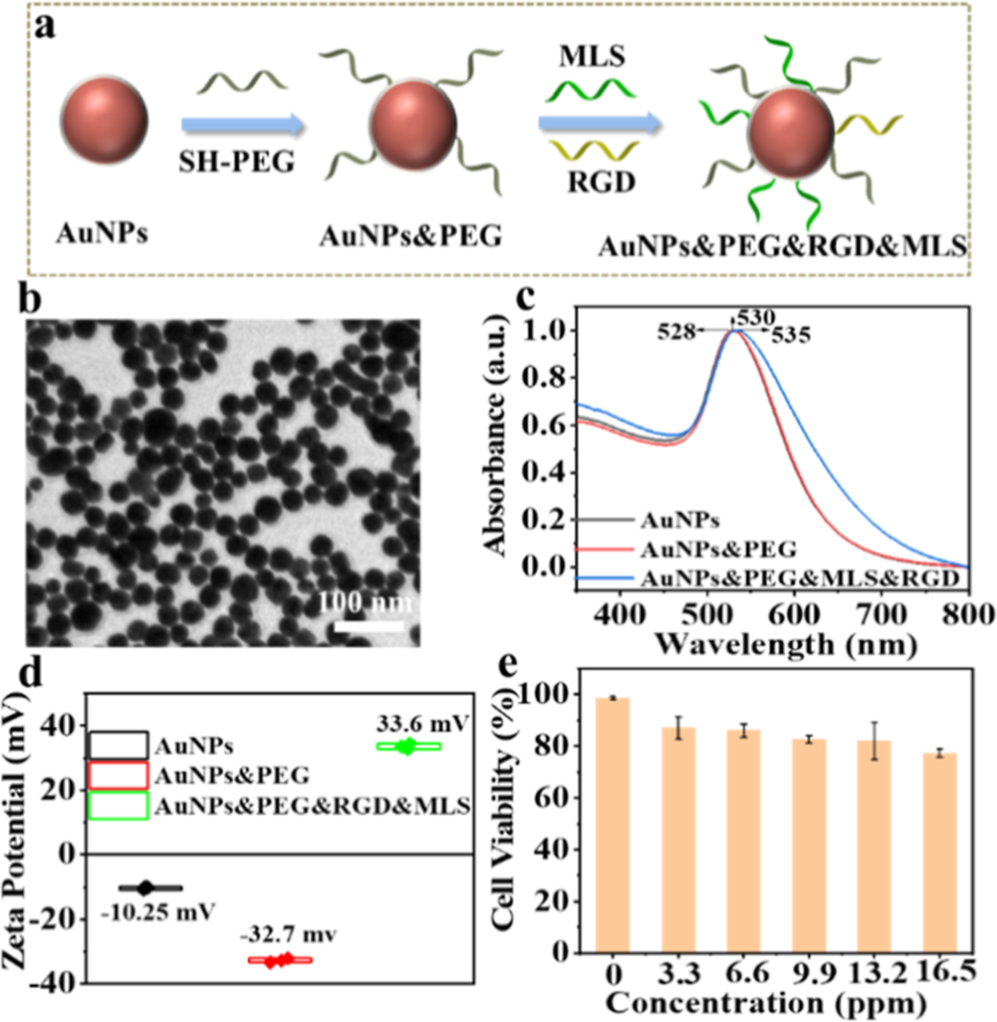
(a) Schematic diagram of preparation mitochondrial targeting nanoprobes. (b) TEM image of AuNPs. (c,d) UV–vis spectra and zeta potential of AuNPs, AuNPs&PEG, and AuNPs@PEG&MLS&RGD as the mitochondrial targeting nanoprobe. (e) Cell viability of DPSCs incubated with nanoprobes under different concentrations.

### Revealing Molecular Insights of Mitochondria within DPSCs during Cell Differentiation by Label-Free SERS Spectra

To monitor the molecular insights of mitochondria during cell differentiation, label-free spectra were applied in our work. The mitochondrial targeting nanoprobes (MT-AuNPs) were incubated with DPSCs for 24 h and then stimulated with IES for different days. The good cell viability of DPSC incubated with nanoprobes was observed after treatment with IES for 3 days (Figure S14). Simultaneously, the effect of MT-AuNPs on cell differentiation was explored, as displayed in Figure S15. The no obvious mineralized nodules were observed from DPSCs incubated with MT-AuNPs for 3 days, compared with IES groups. Subsequently, the targeting of nanoprobes to mitochondria was estimated using the Bio-TEM images, as shown in Figure 5a,b. Most of the MT-AuNPs entered into the mitochondria, and the cell and mitochondria still maintained their structure. It suggested that the nanoprobes of MT-AuNPs possessed good targeting to mitochondria and good biocompatibility. Simultaneously, the nanoprobes in the mitochondria have not been degraded after 3 days of incubation. The repetitiveness of nanoprobes for label-free SERS detection was evaluated, as shown in Figure S16. The relative standard deviation (RSD) values of SERS intensity and area at 999 cm^–1^ calculated were 2.63% and 8.5%, respectively. The results demonstrated that this method possesses good repetitiveness due to RSD values less than 20%,^35,36^ which is suitable for the detection of biological systems. Subsequently, the SERS spectra of mitochondria within DPSCs incubated with nanoprobes were collected during the cell differentiation process (Figure 5c), and these were assigned (Table S2). Initially, the −S–S– vibrations were located at 495 cm^–1^,^26^ the SERS intensity and area of which were slightly reduced with the extension of the differentiation time (Figure 5d,e). The −C–S– vibration was located at 647 cm^–1^,^37^ which was obviously elevated during the cell differentiation process (Figure 5d). It is consistent with the variation trend of the SERS area, as shown in Figure 5e. It indicated that the expression levels of proteins containing −C–S– were gradually boosted with cell differentiation prolonged. Notably, the benzene ring stretching vibration of phenylalanine was observed at 999 cm^–1^,^38^ the content of which within mitochondria was visibly consumed after DPSC differentiation by IES for 1 day. Interestingly, the glycogen vibration was observed from the Raman band at 1145 cm^–1^, the SERS intensity and area of which increased over differentiation time. It manifested that the contents of glycogen within mitochondria were boosted with the differentiation time extended, which is conducive to promoting ATP generation to further accelerate cell differentiation. Additionally, the band at 1564 cm^–1^ was assigned to tryptophan,^39^ which played an important role in regulating the cell differentiation,^40^ the SERS intensity, and the area of which were obviously raised on the second day of cell differentiation (Figure 5d,e). The expression levels of tryptophan in mitochondria also gradually increased under the differentiation stage. It implies some endogenous tryptophan biosynthesis occurred to promote the synthesis of related proteins to accelerate DPSC differentiation. While the level of tryptophan within cells was reduced at 3 days, the reason for which may be the synthesis of functional proteins restraining the tryptophan formation.

**Figure 5:**
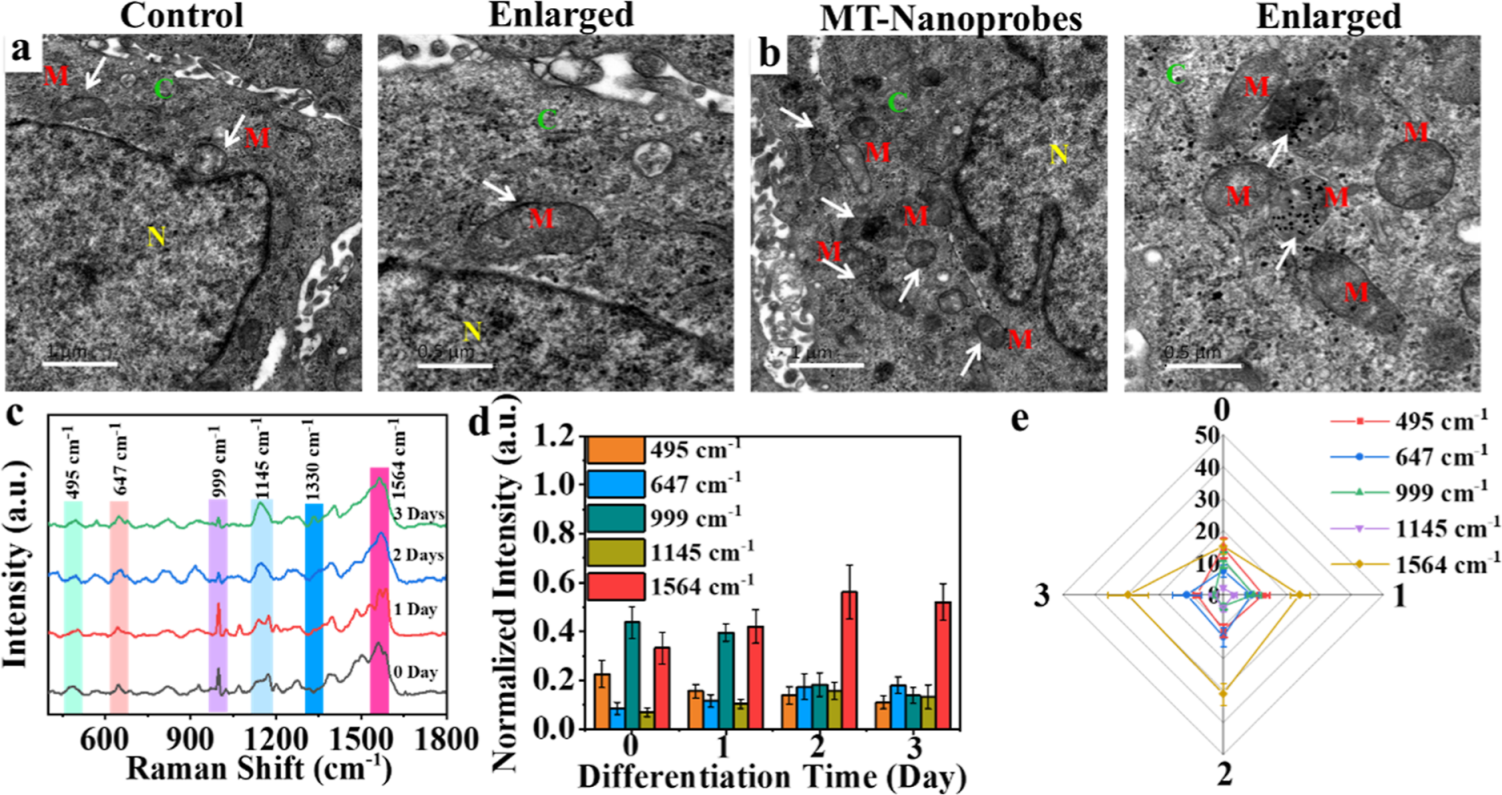
(a) Bio-TEM image and local enlarged image of DPSCs without any treatment. (b) Bio-TEM image and local enlarged image of DPSCs incubated with MT-AuNPs for 3 days. White arrows point to the position of the mitochondria. (M = mitochondria; C = cytoplasm; and N = cell nucleus) The scale bar is 1 μm. The scale bar of enlarged Bio-TEM images is 0.5 μm. (c) Averaged SERS spectra of mitochondria within DPSCs during the cell differentiation process induced by IES at 0.8 V for 5 min per day (impulse widths = 20 s). Each SERS spectrum was averaged from ten SERS spectra. (d,e) The normal SERS intensity and SERS area-related SERS bands at 495, 647, 999, 1145, and 1564 cm^–1^.

## Conclusions

In this work, we propose a highly effective stimulation platform for regulating rapid DPSC differentiation based on IES. This approach can greatly shorten the DPSC differentiation time from usually more than 20 days to only about 3 days, compared with traditional methods such as drug stimulation. Simultaneously, the method developed provides good biocompatibility, controllability, and repeatability. The IES can promote the DPSC differentiation toward the odontogenic direction, which is conducive to the generation of teeth. However, this approach has some disadvantages, such as a lack of feasible options to introduce regenerative treatment to clinical teeth, which may require the development of feasible instruments and wearable patches, and solving this issue requires us to ponder in the future. In addition, IES can promote MMP within DPSCs elevated to contribute to the ATP generation, which further accelerates the rapid DPSC differentiation. The expression levels of mitochondrial fusion proteins within DPSCs are down-regulated during the cell differentiation process. Importantly, the associated molecular events of mitochondria were revealed by label-free SERS spectra. The tryptophan contents within mitochondria were gradually boosted, while the expression levels of phenylalanine were obviously reduced during the differentiation process by IES. This work broadens our perception of dental pulp stem cell differentiation, and this platform proposed provides new possibilities for dental pulp stem cell differentiation to further promote tooth tissue regeneration in the future clinic.

## Supplementary Material



## Notes

The supporting materials are included The Supporting Information is available free of charge on the ACS Publications website. The Supporting Information is available free of charge at https://pubs.acs.org/doi/10.1021/acsmeasuresciau.5c00005.

Experimental part; impulse current–time curves; fluorescence imaging of DPSCs stained with AM/PI; bright-field images of DPSCs stained with alizarin; charge-time profiles; Ca^2+^ and ALP fluorescence images; length distributions of DPSCs; ratio of mean fluorescence intensity of aggregated to monomer (IM); immunofluorescence imaging of Mfn-2; size distribution of AuNPs; and SERS spectra of DPSCs incubated with MT-AuNPs before IES (PDF)
