# Supplementary material for: Molecular Stress Response of Mitochondria during Electrostimulation Evoking Stem Cell Differentiation Revealed by Fluorescence Imaging Combined with SERS Spectra
Source: ACS Meas Sci Au. 2025 Mar 12;5(3):294–303. doi: 10.1021/acsmeasuresciau.5c00005 (PMC12183591; doi:10.1021/acsmeasuresciau.5c00005)
Supplement: Supplementary file 1 [file tg5c00005_si_001.pdf]

# Supporting Information

## Molecular Stress Response of Mitochondria During Electrostimulation Evoking Stem Cell Differentiation Revealed by Fluorescence imaging Combined with SERS Spectra


Jiafeng Wang[1, 4#], Xiaozhang Qu[3#], Zhimin Zhang[1], Xiuping Meng[1], Guohua Qi[2,4]*

[1] Department of Endodontics, Hospital of Stomatology, Jilin University, Changchun 130021, Jilin, P.R. China.

[2] Guangdong Key Laboratory of Biomedical Measurements and Ultrasound Imaging, School of Biomedical Engineering, Shenzhen University Medical School, Shenzhen University, Shenzhen, 518060, China

[3] The First Hospital of Jilin University, Changchun, 130031, P. R. China

[4] State Key Laboratory of Electroanalytical Chemistry, Changchun Institute of Applied Chemistry, Chinese Academy of Sciences, Changchun 130022, Jilin, P. R. China

# Jiafeng Wang and Xiaozhang Qu contributed equally to this article.

* Corresponding Author：ghqi@szu.edu.cn

**Table of Contents**

1. Experimental section……S-4
2. Figure S1. The impulse current-time curves under different impulse voltages after treatment of DPSCs ……S-8
3. Figure S2. The fluorescence imaging of DPSCs stained with AM/PI after impulse ES under different voltages for two days……S-8
4. Figure S3. The bright field images of DPSCs stained with alizarin red after ES in different algebra ……S-8
5. Figure S4. The charge-time profiles of cell electrodes after IES at impulse voltage at 0.8 V under different impulse widths for 5 min……S-9
6. Table S1. The methods of regulation for cell differentiation ……S-9
7. Figure S5. The ALP fluorescence imaging within DPSCs after IES treatment at 0.8 V for 5 min under different days……S-10
8. Figure S6. The fluorescence imaging of DPSCs stained with Ca2+ assay kit in different algebra……S-10
9. Figure S7. The fluorescence imaging of DSPP within DPSCs in control groups under different days and The fluorescence intensity of DSPP within single-cell calculated in control groups under different days……S-10
10. Figure S8. The length distributions of DPSCs after IES treatment at impulse voltage of 0.8 V for 5 min under different days……S-11
11. Figure S9. The ratio of mean fluorescent intensity of aggregated (IA) to monomer (IM) of JC-1……S-11
12. Figure S10. The fluorescence imaging and The fluorescence intensity of Mfn-1 within DPSCs in control groups under different days……S-12
13. Figure S11. The immunofluorescence imaging of Mfn-2 within DPSCs and cell nucleus stained with DAPI after IES treatment at 0.8 V for 5 min under different days……S-12
14. Figure S12. The fluorescence imaging and The fluorescence intensity of Mfn-2 within DPSCs in control groups under different days……S-13

15. Figure S13. The size distribution of AuNPs……………………………………………………………………………S-13
16. Figure S14.The fluorescence imaging of DPSCs under different conditions for 3 days stained with AM/PI……………………………………………………………………………………………………………………………S-13
17. Figure S15.The bright field images of DPSCs stained with alizarin red……………………………………..S-14
18. Figure S16.The SERS spectra of DPSCs incubated with MT-AuNPs before IES……………………………………………………………………………………………………………………………S-14
19. Table S2. The assignment of SERS bands of mitochondria within DPSCs during differentiation process induced by IES ……………………………………………………………………………………………......S-15
20. References……………………………………………………………………………………………………………………S-16

## Experimental Part

### Instrument

The transmission electron microscope (TEM) images of AuNPs were characterized through Hitachi 600 transmission electron microscope (Hitachi, Japan). The UV-vis spectra were recorded through a UV-2600 spectrophotometer (Shimadzu, Japan). The concentration of the AuNPs was detected by an ICAP 6300 inductively coupled plasma emission spectrometer (Thermo Fisher, USA). The bright and fluorescence images were collected using the inverted DMI6000B microscope (Leica, Germany). The zeta potential of nanoparticles was measured by Zetasizer Nano ZS 90 (Malvern, British). The SERS spectra were recorded through a confocal Raman system (LabRAM ARAMIS, HORIBA Jobin Yvon).

### Extraction of dental pulp stem cell from human teeth

The teeth samples collected were approved by the Ethics Committee of the Stomatological Hospital of Jilin University and the informed consent of the subject. Typically, we collected the complete third-molars of 18-25 years old healthy from the maxillofacial surgery. The teeth were stored in a high-glucose Dulbecco minimum essential medium to collect pulp tissue after thorough disinfection. Subsequently, the teeth were opened under aseptic conditions to remove the pulp tissue and repeatedly rinsed, and cut. The mixed solution was centrifugated at 1000 rpm for 5 min and the sediment was dispersed into 200 μL of type I collagen enzyme (3 mg/mL) to cover the pulp tissue and the pulp tissue was digested at 37 ℃ for 15 min. Subsequently, the solution was centrifugated at 1000 rpm for 5 min to remove the supernatant. Finally, the pulp tissue collected was cultured into complete DMEM medium for 3 days. The cells at passage three (P3) were selected after dilution and purification for this study.

### Detection cell viability of DPSCs after IES

Firstly, the DPSCs were planted on the ITO glasses for incubation for 12 h. After the IES for DPSCs at 0.8 V for 5 min of each day under different days (impulse width=20 s), the DPSCs were stained with the mixed solution contained Calcein AM (2 μM) and propidium iodide (PI, 4 μM) for 20 min. After that, the cells were washed three times using the PBS solution (10 mM, pH=7.4) and observed using Leica DMI6000B microscope with a fluorescence detector with 20× objective.

### Bright Field images of DPSCs stained with alizarin red during differentiation process

Typically, the DPSCs were treated with IES at 0.8 V for 5 min under impulse width of 20 s in different differentiation days. The cells were rinsed using the PBS and then the cells were fixed with paraformaldehyde (4wt %) for 20 min. After that, the cells were washed through the PBS three times. Subsequently, the alizarin red solution (1wt %) was applied for staining with DPSCs for 30 min. Finally, the cells were cleaned using the PBS by three times and observed using the Leica DMI6000B microscope under 10× objective.

**Oil red staining for DPSCs during differentiation process**

To verify whether pulsed electrical stimulation induced dental pulp stem cell differentiation toward to adipogenic direction, the typical Oil red staining was applied in this work. The DPSCs were cleaned using the PBS three times after IES under different days and then fixed using paraformaldehyde (4wt %) for 20 min. Subsequently, the cells were washed using the PBS by three times and stained using the oil red (1wt %) for 40 min. Finally, the cleaned cells were observed under Leica DMI6000B microscope with a detector with 10× objective.

**$Ca^{2+}$ level within DPSCs during differentiation process induce by IES**

To detect the $Ca^{2+}$ level within DPSCs under different differentiation days after IES, the commercialized $Ca^{2+}$ assay kit was used in this study. Firstly, the DPSCs were cultured on the ITO glass and then stimulated through IES at 0.8 V for 5 min under pulse width of 20 s for different days. The cells were cleaned with the PBS and then stained using the $Ca^{2+}$ assay kit for 30 min. Finally, the DPSCs were washed using the PBS and recorded with a fluorescence detector of Leica DMI6000B microscope with 10× objective.

**Detection of dentin sialophosphoprotein (DSPP) within DPSCs during differentiation process**

The DPSCs were treated with IES under different differentiation times and then washed using the PBS three times. The DPSCs were fixed using the 4 wt % paraformaldehyde for 20 min. The cells were cleaned through PBS three times. The Triton-X 100 (1wt %) was used to incubate with DPSCs to enhance cell permeability for 20 min. Subsequently, the DPSCs were rinsed with PBS three times and exposed to a blocking buffer (BSA, 1wt %) for 1 h at room temperature. After that, the cleaned cells were incubated with hDSPP antibody labeled on FITC (at a dilution of 2:1000) overnight at 4 ℃. The cell nucleus was dyed using the DAPI (1 μM) for 10 min at room temperature. Finally, the cells were washed using the

PBS three times and collected under the Leica DMI6000B microscope with a fluorescence detector. All the fluorescent intensity of DSPP within cells was calculated using the software of Image-J.

**Detection of mitochondrial transmembrane potential within DPSCs during differentiation process**

Mitochondria membrane potential (MMP) was considered as an important indicator for regulating cell life activities. In this work, we have employed the commercial assay kit of JC-1 to check the MMP changes within DPSCs during cell differentiation process induced by IES. The cells were cleaned using PBS three times after treatment by IES under different days (0, 1, 2 and 3 days). Then, the cells were stained with commercial dye of JC-1 assay kit for 20 min. Subsequently, the cells were washed through PBS three times and the fluorescence imaging of cells were recorded using Leica DMI6000B microscope with a fluorescence detector with 20× objective (EM: 510-540 nm (JC-1 monomer) and EM: 570-620 nm (JC-1 aggregate).

**Adenosine Triphosphate expression within DPSCs stimulated by IES at different days**

To check the adenosine triphosphate (ATP) level within DPSCs cells, the ATP assay kit was used during the cell differentiation. Briefly, the DPSCs were treated with IES at 0.8 V for 5 min per day under different days (0, 1, 2 and 3 days). The same number of DPSCs was collected to detect the ATP level changes. The cells were collected into centrifuge tubes to remove the supernatant. Afterward, the 200 μL of cell extract solution were added into tubes for ultrasonication in an ice-bath for 1 min. Subsequently, the mixed solution was centrifugated at 6000 rpm in 4 ℃ for 10 min and the supernatant was added into another tube. After that, chloroform (50 μL) was added into tubes and adequately shocked to mix well and then the mixed solution was centrifugated at 6000 rpm in 4 ℃ for 3 min. The supernatant was kept to further detection in 4 ℃. The final supernatant was mixed with work solution according to instruction to detect the absorption at 340 nm.

**Preparation of mitochondrial targeting nanoprobes**

The gold nanoparticles (AuNPs) were synthetized according to the method reported [1], previously. Firstly, 1 mL of $HAuCl_4$ (1 wt %) was added into 99 mL of deionized water to stir for boiling. After that the 1.2 mL of sodium citrate (1 wt %) was added into mixed solution to keep boiling for 15 min. Finally, the solution was cooled under room temperature. Subsequently, the AuNPs were used to prepare the mitochondrial targeting nanoprobes based on our method reported [2], previously. 10 mL of AuNPs solution was mixed with the mPEG-SH (30 μL, 1 mM) for 6 h. After that, the mixed solution was

centrifugated at 6000 rpm for 10 min to remove the remaining ligands. Subsequently, 2 μL of cell-penetrating peptide RGD (RGDRGDRGDRGDPGC, 5 mM) and 20 μL of mitochondria localization signal (MLALLGWWWFFSRKKC, 5 mM) were added into the PEG-AuNPs solution to stir for 12 h. Finally, the mixed solution was centrifugated at 6000 rpm for 10 min and cleaned using the water. The nanoprobes were stored at 4℃ for further using.

**Biocompatibility of mitochondrial targeting nanoprobes**

To validate the biocompatibility of mitochondrial targeting nanoprobes for DPSCs, the standardized MTT assay kit was performed. The DPSCs ($5.0\times10^3$ cells in each well) were planted in a 96-hole plate for 24 h and then cleaned using the PBS by three times. Subsequently, the cells were incubated with mitochondria-targeting nanoprobes for 24 h and then washed by PBS three times to remove residual nanoprobes. After that, 10 μL of MTT solution (5 mg/mL) was added into each well to incubate at 37 °C for another 4 h. Finally, 150 μL of DMSO after removing the supernatant medium was added into each well after reaction to dissolve the purple formazan crystals formed. The absorbance values of the wells were measured using on a microplate reader at 570 nm.

**SERS spectra of mitochondria within DPSCs after IES treatment under different days**

Typically, the DPSCs were cultured on the ITO glass for 12 h and then cleaned using the PBS three times. After that, the cells were incubated with mitochondrial targeting nanoprobes (13.2 ppm) for 12 h. The cells were washed using the PBS three times and then stimulated with IES at 0.8 V for 5 min under pulse width of 20 s per day under different days (0, 1, 2, and 3 days). Subsequently, the cells were rinsed through PBS three times and the molecular profiles of mitochondria during cell differentiation were collected using a confocal Raman spectrometer. The excitation wavelength was set as 632.8 nm with 7.1 mW of laser power. The Raman scattering light collection and laser excitation were through a 50× microscope objective lens. The spectral range from 400 to 1800 $cm^{-1}$ was collected with an integration time of 10 s and one accumulation.

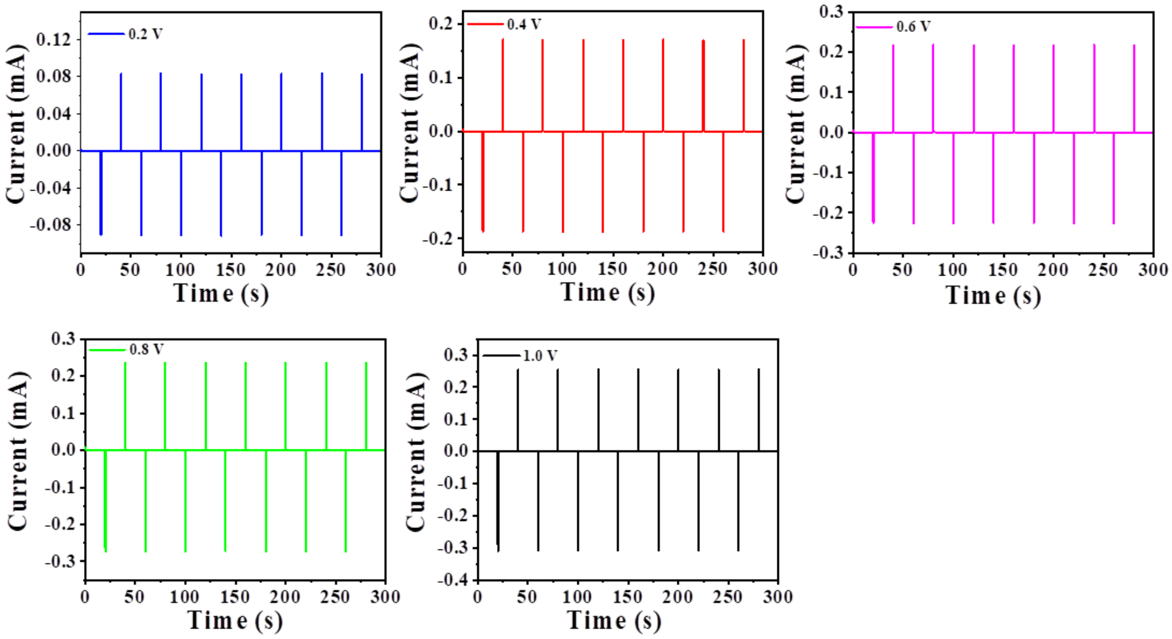


**Figure S1.** The impulse current-time curves under different impulse voltages after treatment of DPSCs. (impulse width=20 s)

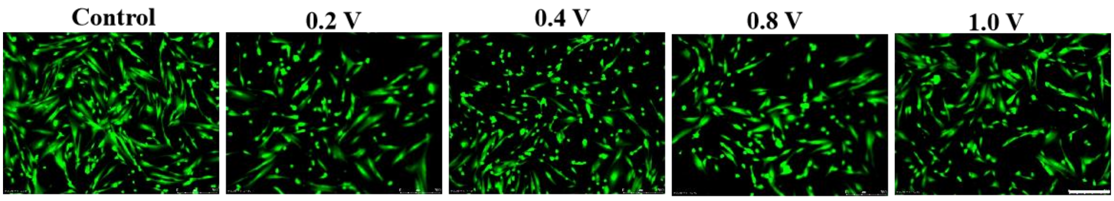


**Figure S2.** The fluorescence imaging of DPSCs stained with AM/PI after IES under different voltages for two days. The scale bar is 250 μm.

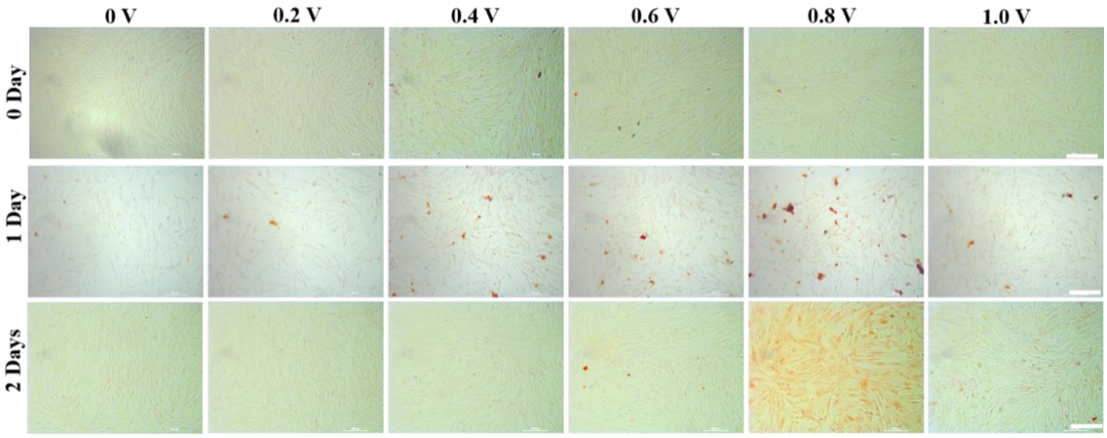


**FigureS3.** The bright field images of DPSCs stained with alizarin red after ES under different impulse voltages for different days. The scale bar is 200 μm.

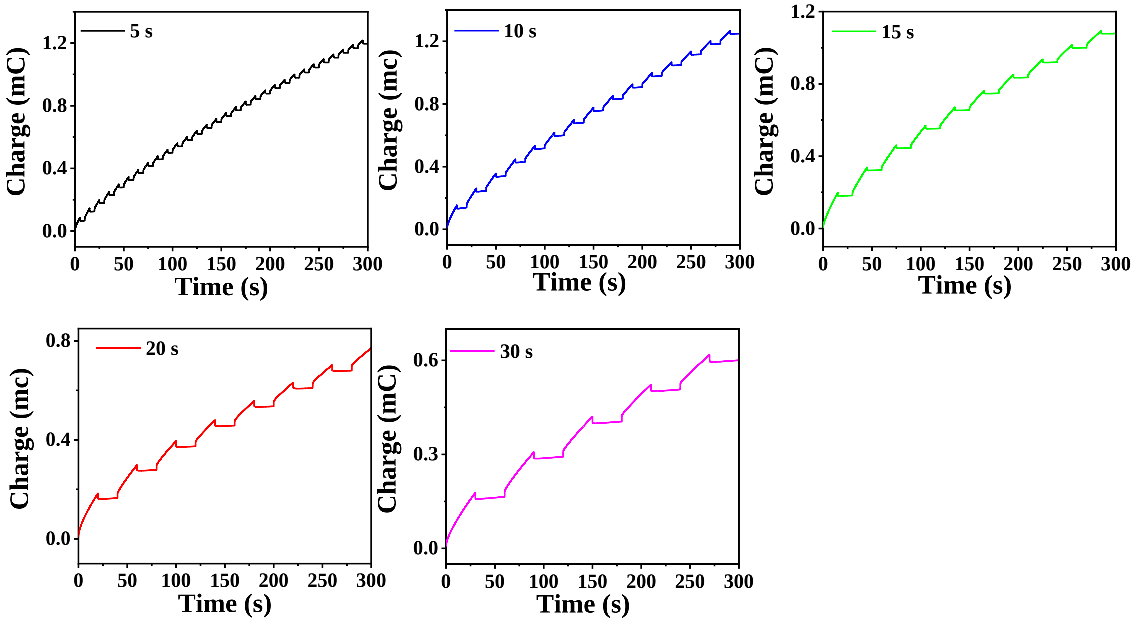


**FigureS4.** The charge-time profiles of cell electrodes after IES at impulse voltage at 0.8 V under different impulse widths for 5 min.

**Table S1.** The methods of regulation for cell differentiation[3-8]

| No. | Method | Time | Cell lines | Reference |
|---|---|---|---|---|
| 1 | Drug | 21 days | DPSCs | Biomaterials 2021,275,120969 |
| 2 | Nanopit Arrays | 14 days | NSCs | Sci. Adv.2022, 8, eabj7736 |
| 3 | Micropatterns | 14 days | hMSCs | Nano Lett.2015, 15, 1457. |
| 4 | NanoScript | 7 days | MSCs | ACS NaNO 2015,7, 6909. |
| 5 | AgNPs | 5 days | ESCs | ACS NaNO 2019,13, 2050. |
| 6 | Thermoplasmonic | 5 days | DPSCs | Anal. Chem. 2022, 94, 9564 |
| 7 | IES | 3 days | DPSCs | Our work |

**Note:** Human Dental pulp stem cell (DPSCs); Neural stem cells (NSCs); human mesenchymal stem cells (hMSCs); Mesenchymal stem cells (MSCs); Embryonic stem cells (ESCs); Impulse electrical stimulation (IES).

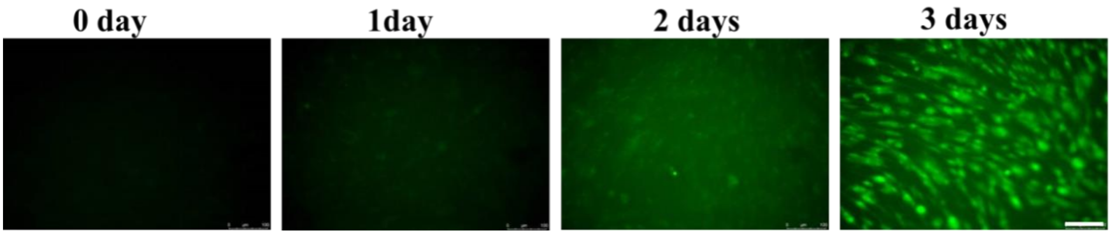


**Figure S5.** The ALP fluorescence imaging within DPSCs after IES treatment at 0.8 V for 5 min under different days (impulse widths=20 s). The scale bar is 100 μm.

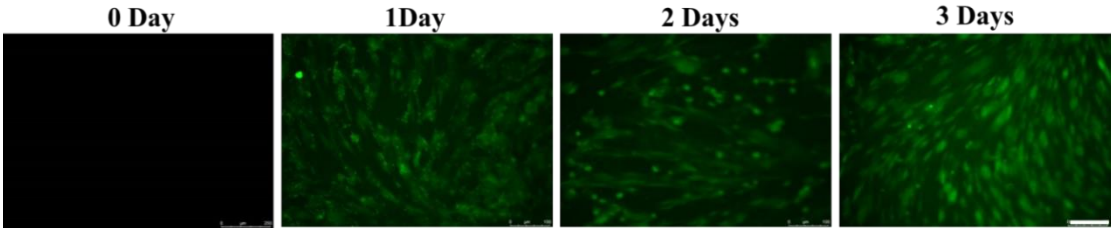


**Figure S6.** The fluorescence imaging of DPSCs stained with $Ca^{2+}$ assay kit after impulse ES treatment at 0.8 V for 5 min under different days (impulse widths=20 s). The scale bar is 100 μm.

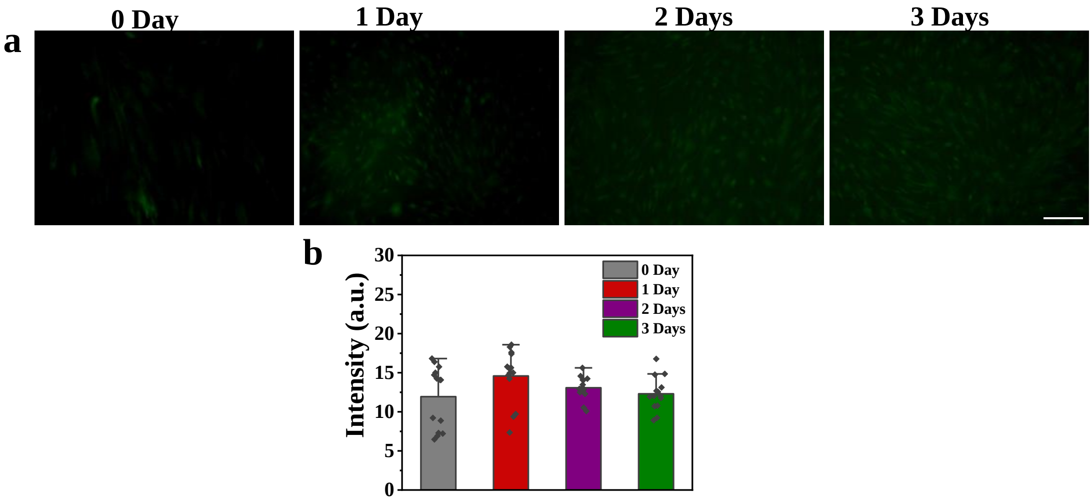


**Figure S7.** (a) The fluorescence imaging of DSPP within DPSCs in control groups under different days (0, 1, 2 and 3 days), The scale bar is 100 μm. (b) The fluorescence intensity of DSPP within single-cell calculated in control groups under different days.

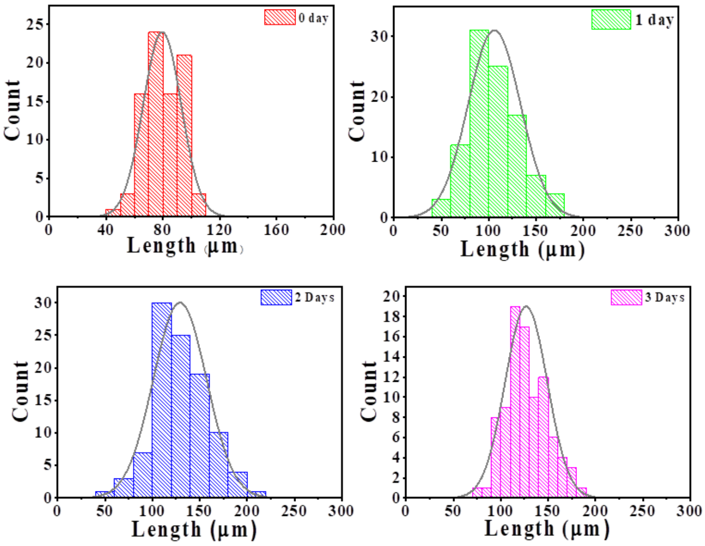


**Figure S8.** The length distributions of DPSCs after IES treatment at impulse voltage of 0.8 V for 5 min under different days. (impulse width=20 s)

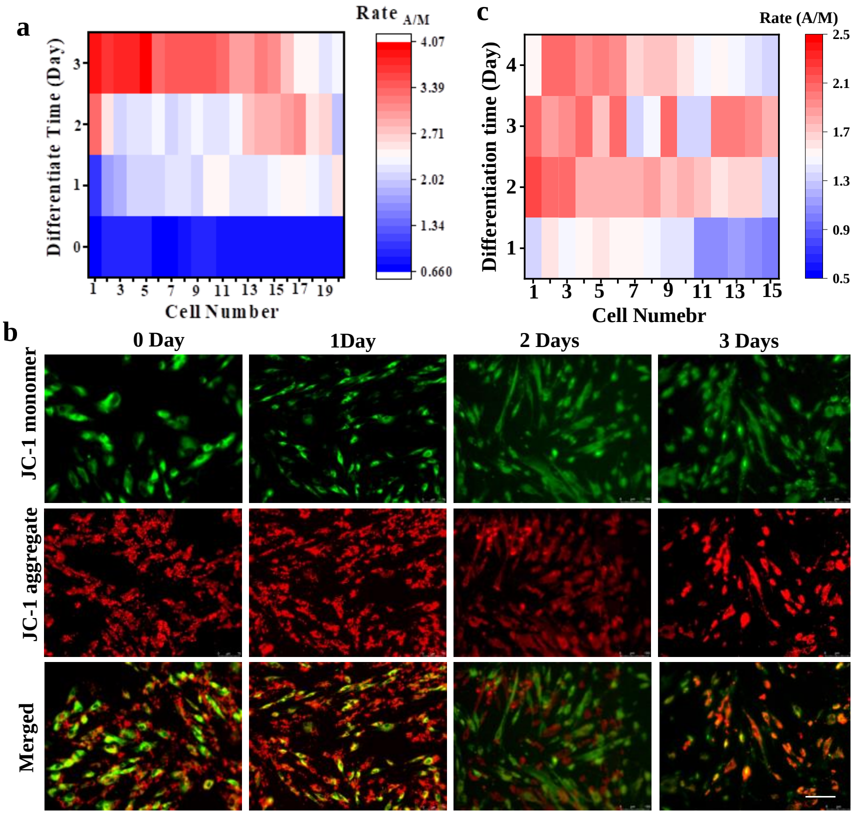


**Figure S9.** The ratio of mean fluorescent intensity of aggregated (IA) to monomer (IM) of JC-1 obtained from 15 single cells of DPSCs during different days in control groups (a) and IES groups (c). (b) The fluorescence images of MMP within DPSCs cells in control groups after stained with JC-1 assay kit during different days (0, 1, 2, and 3 days). The scale bar is 100 μm.

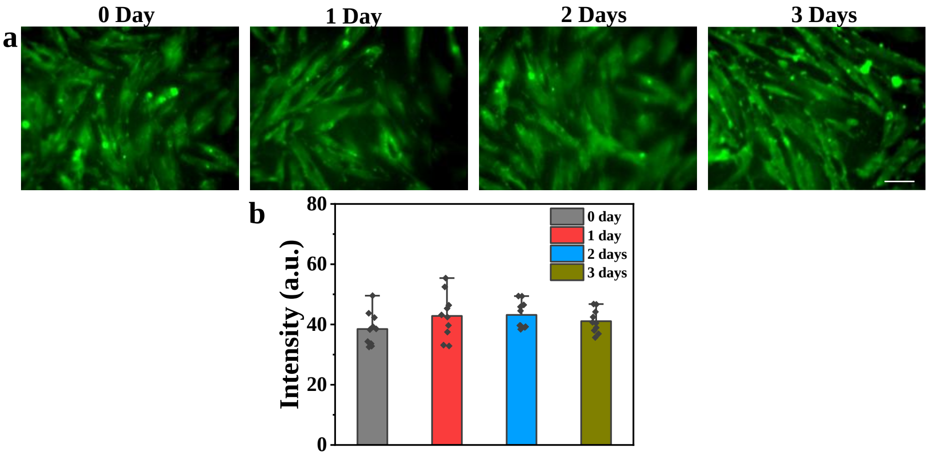


**Figure S10.** (a) The fluorescence imaging of Mfn-1 within DPSCs in control groups under different days. The scale bar is 50 μm. (b) The fluorescence intensity of Mfn-1 within DPSCs in control groups during different days.

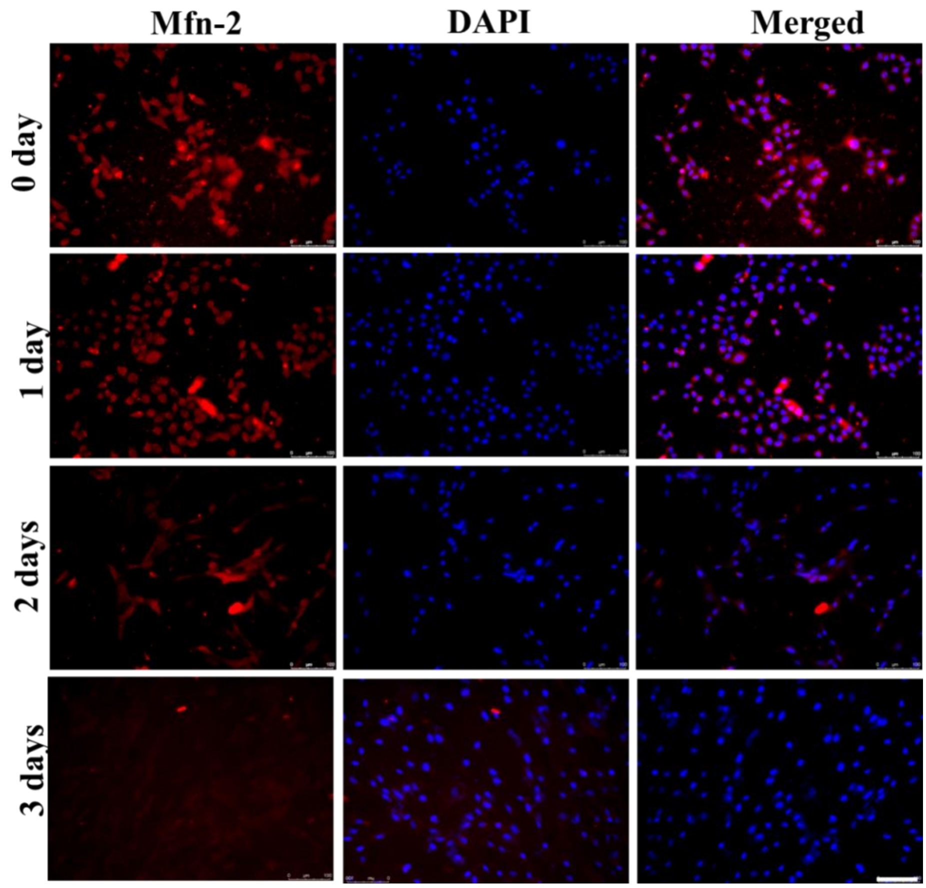


**Figure S11.** The immunofluorescence imaging of Mfn-2 within DPSCs and cell nucleus stained with DAPI after IES treatment at 0.8 V for 5 min under different days (impulse widths=20 s). The scale bar is 100 μm.

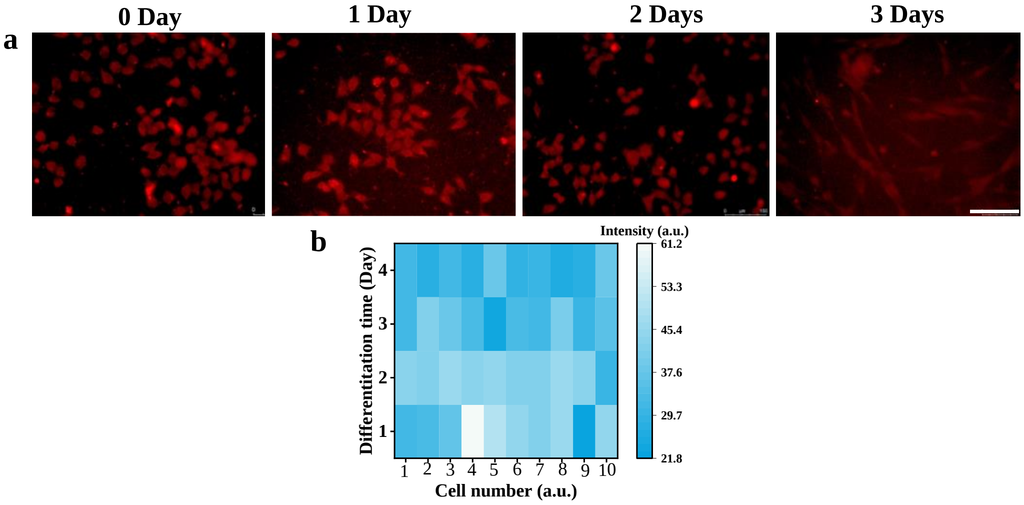


**Figure S12.** (a) The fluorescence imaging of Mfn-2 within DPSCs in control groups under different days. The scale bar is 100 µm. (b) The fluorescence intensity of Mfn-2 within DPSCs in control groups during different days.

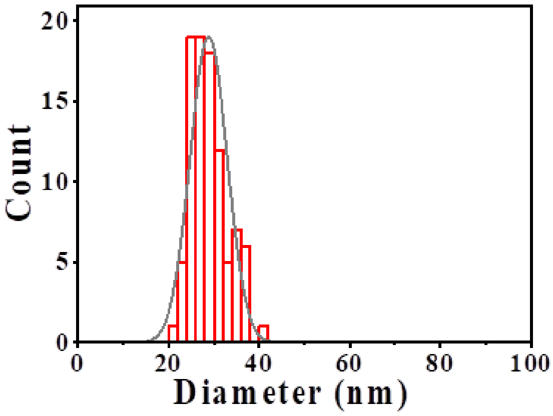


**Figure S13.** The size distribution of AuNPs.

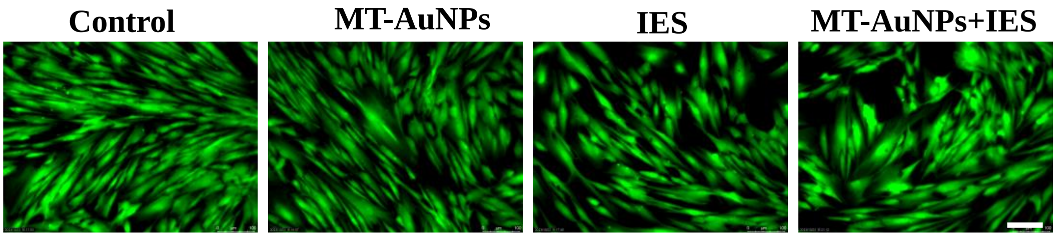


**Figure S14.**The fluorescence imaging of DPSCs under different conditions for 3 days stained with AM/PI. The scale bar is 100 µm.

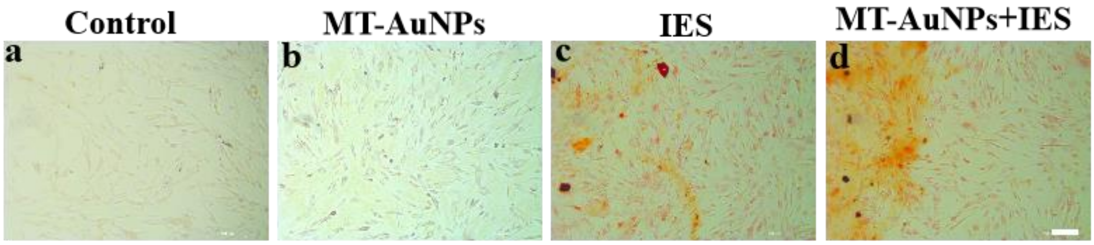


**Figure S15.** The bright field images of DPSCs stained with alizarin red. (a) control group without any treatment; (b)The DPSCs incubated with MT-AuNPs for 3 days; (c) and (d) DPSCs incubated without/ with MT-AuNPs and treated with IES at 0.8 V for 5 min (impulse width=20 s) per day for 3 days. The scale bar is 200 μm.

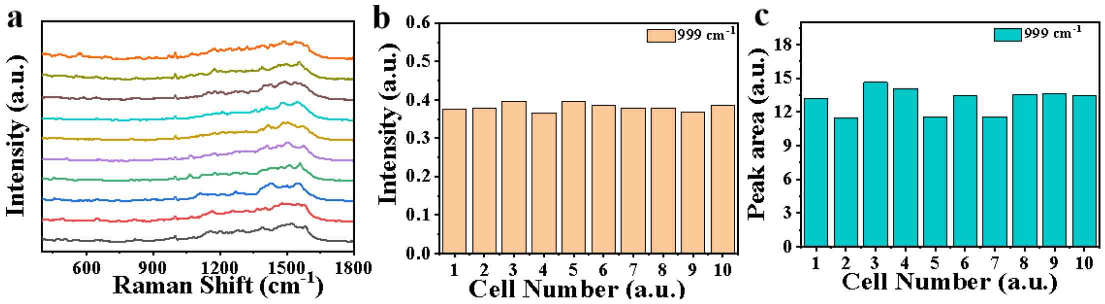


**Figure S16.** (a)The SERS spectra of DPSCs incubated with MT-AuNPs before IES. (b)-(c) The normalized SERS intensity and peak area at 999 $cm^{-1}$ calculated from different cells.

**Table S2.** The assignment of SERS bands of mitochondria within DPSCs during differentiation process induced by IES.[9-12]

| Raman Shift ($cm^{-1}$) Cell differentiation time (Day) | | | | Assignment | Component |
|---|---|---|---|---|---|
| 0 | 1 | 2 | 3 | | |
| 491 | 498 | 492 | 495 | -S-S | Protein |
| 570 | 567 | 570 | 570 | Tryptophan | Protein |
| 644 | 647 | 647 | 648 | -C-S | Protein |
| 737 | 737 | 740 | 742 | T | DNA |
| 820 | 820 | 820 | 820 | Phosphodiester | DNA |
| 927 | 922 | 920 | 922 | Glucose | Carbohydrate |
| 999 | 999 | 999 | 999 | Phenylalanine | Protein |
| 1025 | 1023 | | | -C-H- of phenylalanine | Protein |
| | | 1050 | 1055 | C-O, C-N stretching | Protein |
| 1071 | 1071 | | | Carbonate symmetric stretching | Carbohydrate |
| 1145 | 1145 | 1145 | 1145 | Glycogen | Carbohydrate |
| 1174 | 1174 | | | Tyrosine | Protein |
| 1200 | 1202 | | | C-O, C-N | DNA |
| 1274 | 1270 | 1270 | 1277 | Amide Ⅲ (α-helix) | Protein |
| | | | 1330 | A | DNA |
| 1396 | 1397 | 1396 | 1405 | Glutamate | Protein |
| 1480 | 1482 | 1479 | 1477 | G | DNA |
| 1501 | | | | C | DNA |
| 1560 | 1562 | 1566 | 1564 | Tryptophan | Protein |
| 1590 | 1590 | | | Hydroxyproline | Protein |

**Reference**


1.Turkevich, J.; Stevenson, P. C. Hillier, A Study of the Nucleation and Growth Processes in the Synthesis of Colloidal Gold. *J. Discuss. Faraday Soc.***1951**,*11*, 55-75.

2.Qi, G. H.; Wang, B.; Zhang, Y.; Li, H. J.; Li, C. P.; Xu, W. Q.; Jin, Y. D. Living-Cell Imaging of Mitochondrial Membrane Potential Oscillation and Phenylalanine Metabolism Modulation during Periodic Electrostimulus. *Anal. Chem.* **2019**, *91*, 9571-9579.

3. Ehlinger, C.; Mathieu, E.; Rabineau, M.; Ball, V.; Lavalle, P.; Haikel, Y.; Vautier, D.; Kocgozlu, L. Insensitivity of Dental Pulp Stem Cells Migration to Substrate Stiffness. *Biomaterials* **2021**, *275*, 120969.

4. Cho, Y. W.; Jee, S.; Suhito, I, R.; Lee, J. H.; Park, C. G.; Choi, K. M.; Kim, T. H. Single Metal-Organic Framework-Embedded Nanopit Arrays: A New Way to Control Neural Stem Cell Differentiation. *Sci. Adv.* **2022**, *8*, eabj7736.

5.Wang, X.; Li, S.Y.; Yan, C.; Liu, P.; Ding, J.D. Fabrication of RGD Micro/Nanopattern and Corresponding Study of Stem Cell Differentiation. *Nano Lett.* **2015**, *15*, 1457-1467.

6. Patel, S.; Yin, P. T.; Sugiyama, H.; Lee, K. B. Inducing Stem Cell Myogenesis Using NanoScript. *ACS Nano*, **2015**, *9*, 6909-6917.

7.Zhang, J.; Chen, Y. J.; Gao, M.; Wang, Z.; Liu, R.; Xia, T.; Liu, S. J. Silver Nanoparticles Compromise Female Embryonic Stem Cell Differentiation through Disturbing X Chromosome Inactivation. *ACS Nano*, **2019**, *13*, 2050-2061.

8.Wang, J. F.; Qu, X. Z.; Xu, C.; Zhang, Z. M.; Qi, G. H.; Jin, Y. D. Thermoplasmonic Regulation of the Mitochondrial Metabolic State for Promoting Directed Differentiation of Dental Pulp Stem Cells. *Anal. Chem.* **2022**, *94*, 9564-9571.

9.Stone, N.; Kendall, C.; Smith, J.; Crow, P.; Barr, H. Raman spectroscopy for identification of epithelial cancers, *Faraday Discuss.* **2004**, *126*, 141-157.

10.Movasaghi, Z.; Rehman, S.; Rehman, I. U. Raman Spectroscopy of Biological Tissues. *Appl. Spectrosc. Rev*. **2007**, *42*, 493-541.

11.Aioub, M.; El-Sayed, M. A. A Real-Time Surface Enhanced Raman Spectroscopy Study of Plasmonic Photothermal Cell Death Using Targeted Gold Nanoparticles. *J. Am. Chem. Soc.* **2016**, *138*, 1258-1264.

12.Qi, G. H.; Zhang, Y.; Xu, S. P.; Li, C. P.; Wang, D. D.; Li, H. J.; Jin, Y. D. Nucleus and Mitochondria Targeting Theranostic Plasmonic Surface-Enhanced Raman Spectroscopy Nanoprobes as a Means for Revealing Molecular Stress Response Differences in Hyperthermia Cell Death between Cancerous and

Normal Cells. *Anal. Chem.* **2018**, *90*, 13356-13364.
